# Coevolution unveiled: Sulfate transporters mediate rice resistance and susceptibility to *Xanthomonas oryzae* pv. *oryzicola*

**DOI:** 10.1111/pbi.14377

**Published:** 2024-06-03

**Authors:** Muhammad Sohaib Shafique, Liu Yapei, Li Man, Wang Hongjie, Su Ruyi, Wang Chunlian, Ji Zhiyuan

**Affiliations:** ^1^ State Key Laboratory of Crop Gene Resources and Breeding/ National Key Facility for Crop Gene Resources and Genetic Improvement, Institute of Crop Sciences Chinese Academy of Agricultural Sciences Beijing China

**Keywords:** bacterial leaf streak, natural variation, effector‐binding element, designer TALE, resistance

Dear Editor,

Rice (*Oryza sativa* L.) is an important cereal crop, feeding more than half of the world's population. The pathogen *Xanthomonas oryzae* pv. *oryzicola* (*Xoc*) causes bacterial leaf streak (BLS) in rice and poses an emerging threat to food security (Liu *et al*., 2014). Genetic resistance is an efficient and sustainable solution to control diseases. Resistance resources against *Xoc* are scarce and largely unexplored, seeking uncontrolled pathogen attacks, hindering BLS control. Moreover, *Xoc* virulence is dependent upon the exploitation of susceptibility (*S*) factors to cause disease (Cernadas *et al*., 2014). Therefore, research exploring natural sources of resistance and understanding genetic susceptibility in the rice‐*Xoc* interaction is critical for controlling the spread of BLS.

The virulence of *Xoc* depends on injecting transcription activator‐like effectors (TALEs) into rice cells, which then bind to effector‐binding elements (EBEs) in the promoter region to stimulate gene expression. This transcriptional reprogramming typically targets *S* genes, which facilitate pathogen proliferation and promote disease susceptibility. Natural variation in the *S* gene is a potent but currently unexplored resource for resistance against BLS. Genes such as *xa13*, *xa25* and *xa41* are allelic variants of sugar transporter (OsSWEET) genes, harbouring polymorphic EBE sites for the bacterial blight pathogen *X. oryzae* pv. *oryzae* (*Xoo*), rendering TALEs unable to induce gene expression, thus conferring broad‐spectrum but recessive resistance against *Xoo* (Blanvillain‐Baufume *et al*., 2017; Oliva *et al*., 2019). One member of the **Sul**fate **tr**ansporter (SULTR) family, *OsSULTR3;6*, serves as the *S* gene when induced by the TalBF TALE family of *Xoc* (Table S2) . To date, there have been no reported instances of natural *S* gene variation conferring resistance against *Xoc*.

To mine novel allelic variations within *OsSULTR3;6* and its promoter, we initiated an exploration of the 3000 Rice Genomes Project data. We identified a total of 43 indels in the promoter and 171 SNPs along with 62 indels within the gene body region. Interestingly, a variation spanning the EBE region for Tal2g_BLS256_/Tal5d_RS105_ (named ∆EBE_TalBF_ hereafter) resulted in the deletion of the entire EBE region was identified (Figure 1a). While only 1% of genome‐sequenced cultivars exhibit the ΔEBE_TalBF_ variation, genotyping by sequencing of these cultivars revealed its presence in 47 genotypes (Figure S1; Table S1). To assess ΔEBE_TalBF_ contribution to resistance, four cultivars were inoculated with *Xoc* strain RS105, revealing significantly enhanced resistance at 14 days of post‐inoculation (dpi) (Figure S2). We developed designer TALE (dTALE‐NV1) to induce *OsSULTR3;6* in ΔEBE_TalBF_ cultivars. Introducing dTALE‐NV1 into RS105 and inoculating restored susceptibility in ΔEBE_TalBF_ cultivar. (Figure 1b). These results demonstrate that the presence of ΔEBE_TalBF_ confers resistance, while the activation of *OsSULTR3;6* confers susceptibility against *Xoc*.

**Figure 1:**
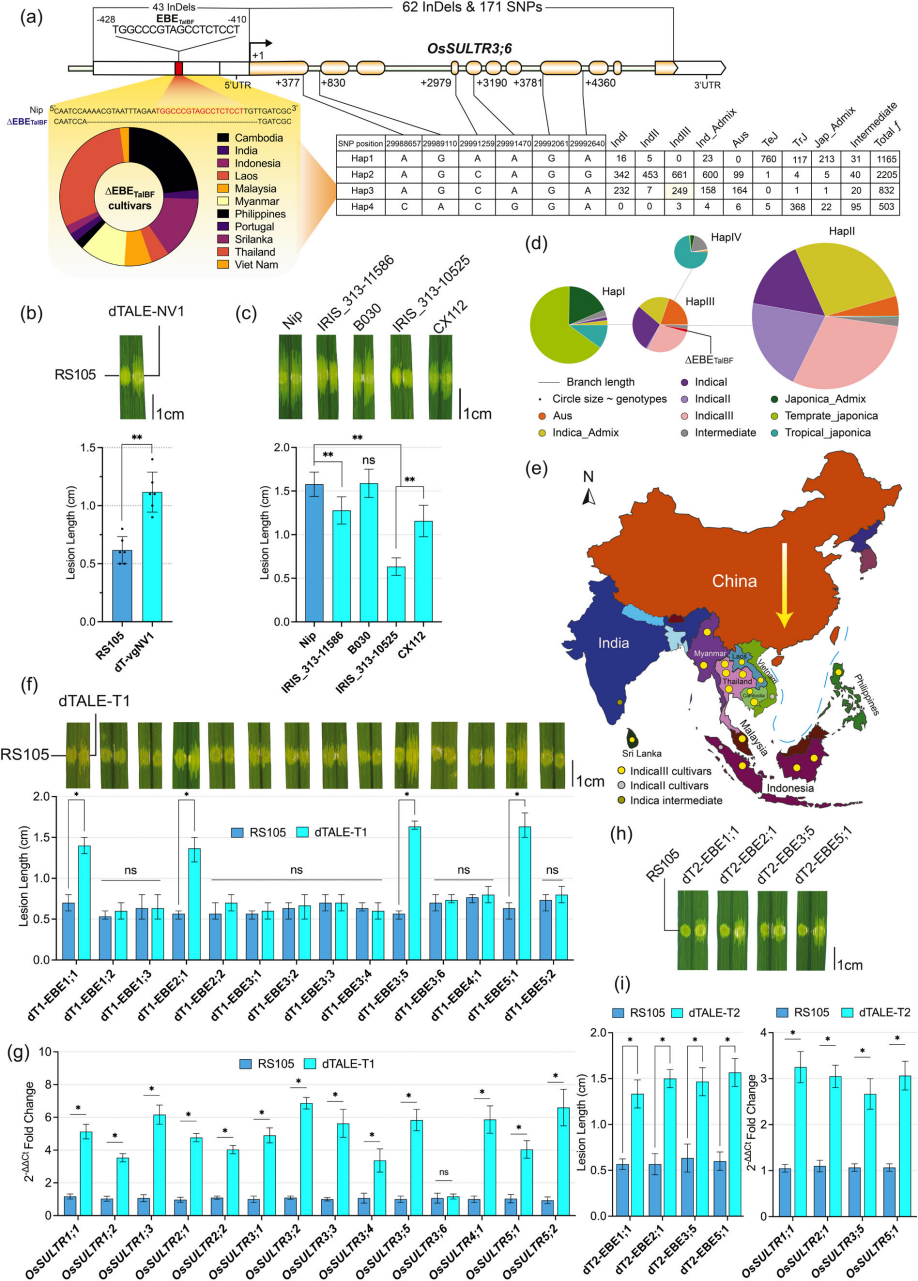
Discovery of novel resistance‐conferring *∆*EBE_TalBF_ variation and four susceptibility supporter genes for *Xoc*. (a) Gene structure, SNP positions and haplotype network analysis of *OsSULTR3;6*, alongside ΔEBE_TalBF_ cultivar sequence. The pie chart displays ∆EBE_TalBF_ cultivar distribution by country. (b) dTALE‐NV1 activation of *OsSULTR3;6* in the ∆EBE_TalBF_ cultivar results in significant lesion expansion, restoring susceptibility. **P<0.01 (Student's *t*‐test). (c) The phenotype of *Xoc* inoculation in cultivars representing each haplotype, with Nip used as control. **P<0.01, ns=no significance (Student's *t*‐test). (d) Contribution of subpopulations in each haplotype, with circle sizes and colours denoting cultivar counts. ∆EBE_TalBF_ cultivars marked in HapIII. (e) The map illustrates the genetic and geographical specificity of ∆EBE_TalBF_ cultivars, primarily belonging to the Ind‐III subpopulation in Southeast Asia. The yellow gradient arrow denotes increasing BLS incidence in China's southern humid regions. (f) The phenotype of lesion length increment after parallel inoculation with dTALE‐T1 and RS105, indicating longer lesion lengths due to activation of four genes. *P<0.05, ns= no significance (Student's t‐test) as compared with RS105 inoculation. (g) Quantitative PCR demonstrates the induction of each SULTR gene by dTALE‐T1 calculated using 2^−ΔΔCT^ method after 24‐h post‐inoculation. (h) Lesion increment phenotype observed after inoculation with dTALE‐T2. Data collected at 10 dpi. (i) Significant differences in lesion size (left) and transcript levels (right) were observed after dTALE‐T2 24 host post‐inoculation.

Next, we analysed *OsSULTR3;6* through haplotype network analysis, focussing on nonsynonymous and splice variant SNPs, revealing four distinct haplotype groups (Figure 1a,d). For resistance phenotyping, cultivars from each haplotype were genotyped and inoculated with RS105; HapIII cultivars had notably smaller lesions, followed by HapIV, HapI and HapII (Figure 1c). Nearly all ΔEBE_TalBF_ cultivars belonged to HapIII, and 98% of them were traced to the Ind‐III subpopulation from Southeast Asia (Figure 1a,e; Table S1). The genetic diversity indexes indicate the minimal abundance of rare *OsSULTR3;6* allele and recent evolution within the Ind‐III subpopulation (Table S5a,b). The regional specificity of ΔEBE_TalBF_ suggests its coevolution and selective sweep in Southeast Asia, where BLS attacks are prevalent.

Genome editing of the EBE in *OsSULTR3;6* confers TALE‐specific resistance against BLS (Xu *et al*., 2021). Besides *OsSULTR3;6*, the role of SULTR family members in rice's susceptibility to *Xoc* remained elusive. Moreover, members of the *S* gene family provide redundant functions, independently sustaining susceptibility (Antony *et al*., 2010). Previously, dTALEs were used to artificially activate OsSWEET genes, revealing three new *S* genes for *Xoo* (Streubel *et al*., 2013). To explore the role of SULTRs in *Xoc* susceptibility, we initially employed CRISPR/Cas9 to edit *OsSULTR3;6* in the Nipponbare (Nip) cultivar. This resulted in two lines: ΔEBE‐E9 with EBE deletion and ΔCDS‐S1 with *OsSULTR3;6* knockout. Inoculating ΔEBE‐E9 and ΔCDS‐S1 with *Xoc* strains BLS256 and RS105 significantly increased resistance compared with Nip at 14 dpi (Figure S3).

Subsequently, we developed dTALEs for all SULTR family members in rice, using previously described methods (Zheng *et al*., 2014). *OsSULTR5;1* and *OsSULTR5;2*, recently renamed *OsMOT1;1* and *OsMOT1;2* due to the absence of the STAS domain, were also targeted by dTALEs (Table S3). The results revealed that artificial induction of *OsSULTR3;5*, *OsSULTR1;1*, *OsSULTR2;1* and *OsSULTR5;1* produced longer lesions under *Xoc* inoculation (Figure 1f). We observed significantly higher transcript levels of all dTALE target genes, indicating dTALE intact functionality (Figure 1g). Our results suggest that dTALEs targeting these four genes increase *Xoc* susceptibility, evidenced by elevated bacterial count (Figure S4). To our knowledge, this susceptibility has not been reported under natural conditions.

To ascertain these findings, another dTALE was developed targeting a different EBE box (T2) for the four new *S* genes (Table S3). Inoculation of dTALE‐T2 RS105 resulted in significantly larger lesions 10 dpi (Figure 1h,i). Transcript levels and CFU counts significantly increased in dTALE‐T2 inoculated leaves (Figure 1i; Figure S5). Suppressing *OsSULTR2;1* transcription boosts resistance to *Xoo* and *Xoc*, emphasizing its role in susceptibility to both pathogens (Yang *et al*., 2022). The 98% protein similarity between *OsSULTR3;5* and *OsSULTR3;6* suggests functional mimicry. While the role of *OsMOT1;1* and *OsSULTR5;1* in *Xoc* susceptibility was not reported. Protein phylogeny among SULTRs is shown in Figure S6.

In conclusion, our research reveals the first coevolutionary resistance resource in rice against *Xoc*. Our research developed *Xoc*‐resistant material, identified new *S* genes and discovered natural variation conferring *Xoc* resistance. Low genetic diversity in *OsSULTR3;6* across sequenced cultivars highlights its conservation and points ∆EBE_TalBF_ recent emergence. This research deepens our knowledge of rice's genetic susceptibility and highlights its strategic defences in coevolutionary contexts.

## Conflict of interest

The authors declare that there are no competing interests.

## Funding

This work was supported by STI 2030—Major Projects (2022ZD0400203), the National Natural Science Foundation of China (32072412), the Youth Innovation Program of Chinese Academy of Agricultural Science (Y2022QC01) and the Innovation Program of the Chinese Academy of Agricultural Sciences to Z.J., C.W. and Y.L.

## Supporting information


**Figure S1–S5** Supplementary Figures.
**Table S1–S6** Supplementary Tables.

## Data Availability

The bacterial strains and materials employed in this study are accessible upon reasonable request from Dr. Ji Zhiyuan.
