# Supplementary material for: Coevolution unveiled: Sulfate transporters mediate rice resistance and susceptibility to Xanthomonas oryzae pv. oryzicola
Source: Plant Biotechnol J. 2024 Jun 3;22(9):2632–4. doi: 10.1111/pbi.14377 (PMC11331776; doi:10.1111/pbi.14377)
Supplement: Supplementary file 1 — Figure S1–S5 Supplementary Figures. Table S1–S6 Supplementary Tables. [file PBI-22-2632-s001.docx]

**Supplementary Information**

**Coevolution Unveiled: Sulfate Transporters Mediate Rice Resistance and Susceptibility to *Xanthomonas oryzae* pv. *oryzicola***

Muhammad Sohaib Shafique^1,2^, Liu Yapei^1,2^, Li Man^1^, Wang Hongjie^1^, Su Ruyi^1^, Wang Chunlian^1,^*, Ji Zhiyuan^1,^*

*Correspondence: Ji Zhiyuan ([jizhiyuan@caas.cn](mailto:jizhiyuan@caas.cn)), Wang Chunlian ([wangchunlian@caas.cn](mailto:wangchunlian@caas.cn)),

**Table of Contents (Short legends)**

**Figure S1.** Genotyping of ∆EBE_TalBF_ cultivars revealed deletion of whole EBE_Tal2g_ in the Indica III genotypes.

**Figure S2.** Natural variation cultivars conferring resistance to *Xoc* inoculation.

**Figure S3.** Genome editing of *OsSULTR3;6* confers resistance against Xoc

**Figure S4a.** The bacterial population count in ∆CDS-S1 mutant plant after inoculation of the first set of dTALE-RS105 strains.

**Figure S4b.** The bacterial population count in ∆CDS-S1 mutant plant after inoculation of the second set of dTALE-RS105 strains.

**Figure S5.** Phylogenetic tree of SULTRs family in Rice based on sequence similarity in protein.

**Supplementary Tables**

**Table S1.** Cultivar IDs, subpopulation, and locations of cultivars showing ∆EBE_TalBF_ natural variation.

**Table S2.** RVD sequence, alignment, and phylogenetic tree of TalBF family in whole genome sequenced strains of *Xoc.*

**Table S3.** Sulfate transporter gene IDs, designer transcription activator-like effector (dTALE) repeat variable di-residue (RVDs), target sequence, and role in susceptibility.

**Table S4.** Primers used in the present study.

**Table S5.** Genetic diversity index, CDS haplotype information, and diversity of *OsSULTR3;6* in 3KRG.

**Table S6.** Table of plasmids and bacterial strains used in the study.

**
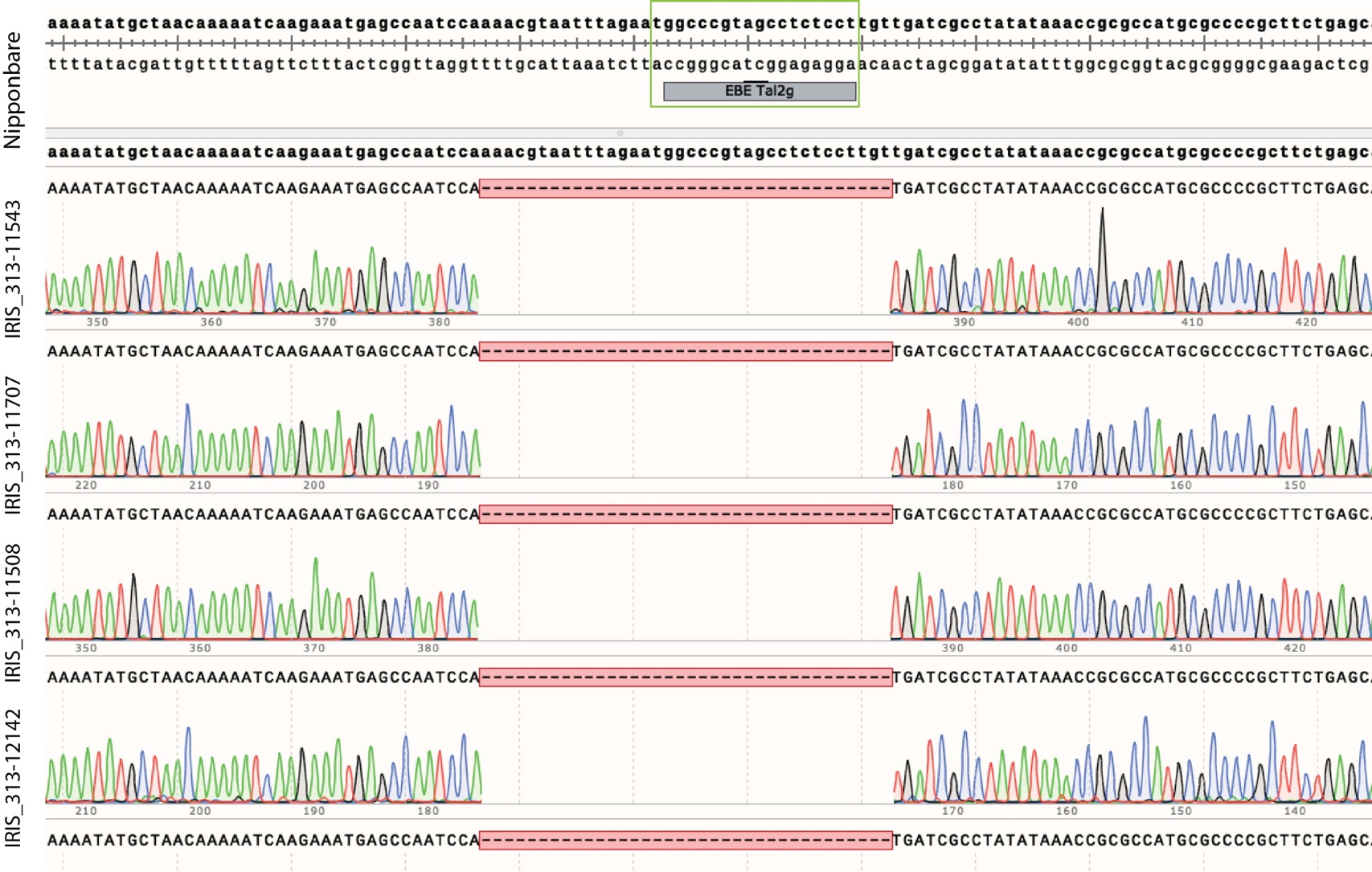
Supplementary Figure 1.** Genotyping of ∆EBE_TalBF_ cultivars revealed deletion of whole EBE_Tal2g_ in the Indica 47 genotypes. Sequencing chromatograms showing the depletion of EBE_TalBF_ and its surrounding base pairs in four cultivars. We genotyped several genotypes (presented in Table S1) and identified ∆EBE_TalBF_ presence.

**
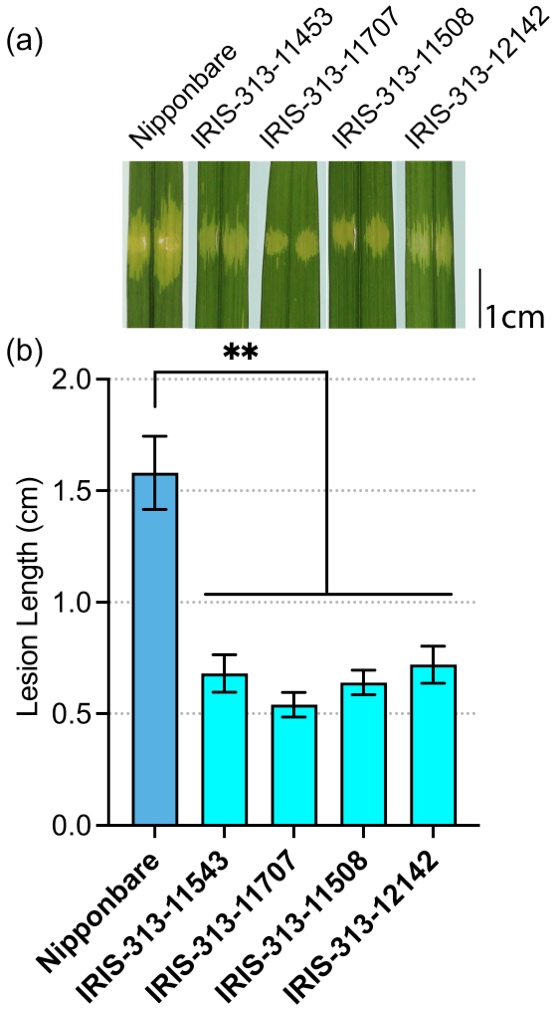
Figure S2.** Natural variation cultivars conferring resistance to *Xoc* inoculation. Inoculation was performed using the RS105 strain of *Xoc*. (a) Resistance phenotype of four selected ∆EBE_TalBF_ cultivars. (b) Lesion length of control Nipponbare and ∆EBE_TalBF_ cultivars. Pictures were taken 14 days post-inoculation. **P<0.01 (Student’s *t*-test).


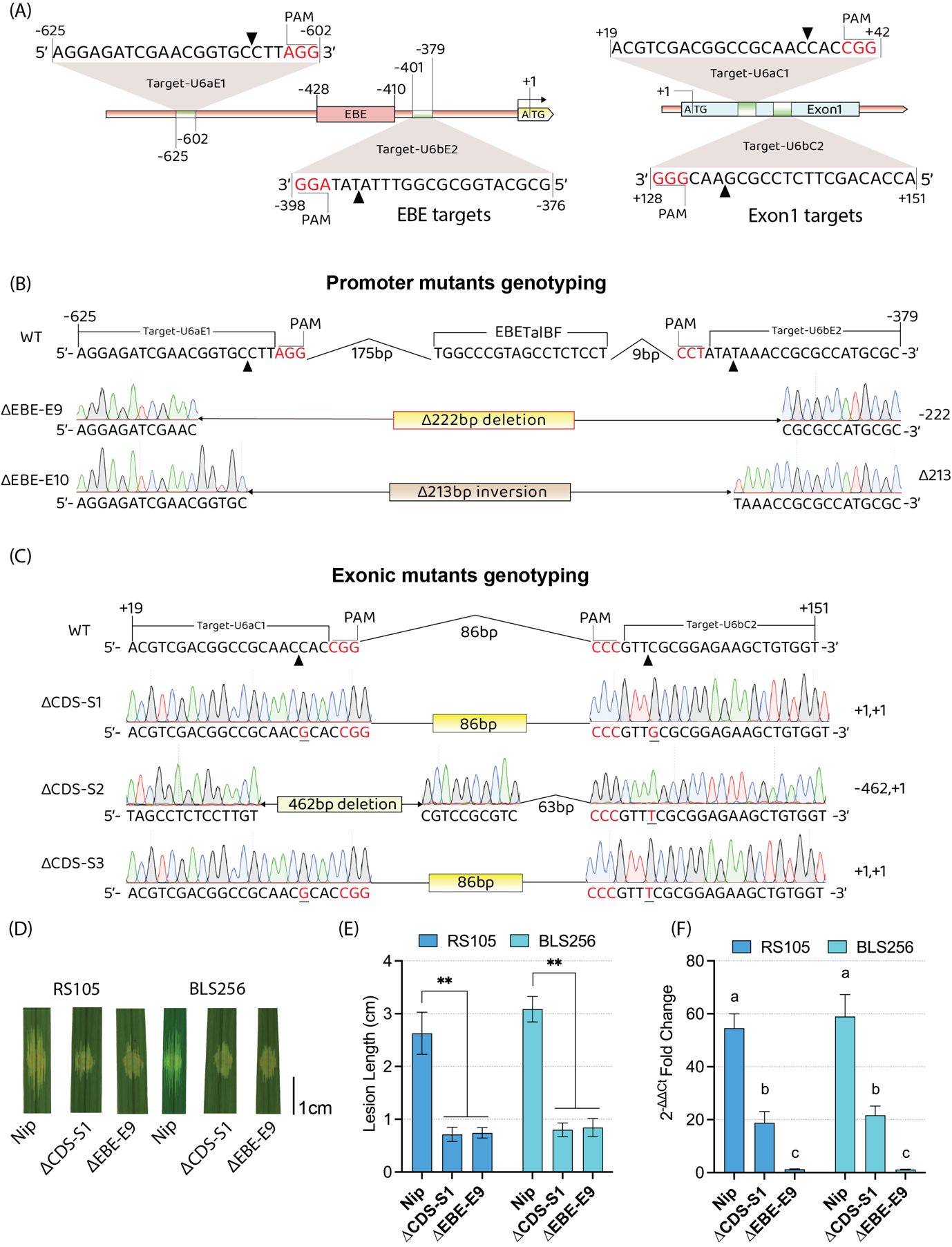


**Figure S3.** Genome editing of *OsSULTR3;6* confers resistance against bacterial leaf streak. (A) Sequence and relative positions of sgRNA target sites from start codon ATG in the promoter spanning EBE (left) and in the first exon of *OsSULTR3;6* (right). The PAM site is shown in red text. Black triangles show the expected double-strand break site. (B) Genotyping by sequencing results of promoter mutant plants showing large deletion of -222 base pairs and segmental inversion. (C) Sequencing results showing gene knockout mutation in exonic mutant plants. Additions are underlined in black. (D) Mutant plants showing resistance to hypervirulent strains RS105 and BLS256. (E) Lesion length of mutant and Nipponbare plant leaves after RS105 and BLS256 inoculation. **P<0.01 (Student’s *t*-test). (D) Quantitative real-time PCR of mutant plants showing significantly lower transcript levels of *OsSULTR3;6* after RS105 and BLS256 ***
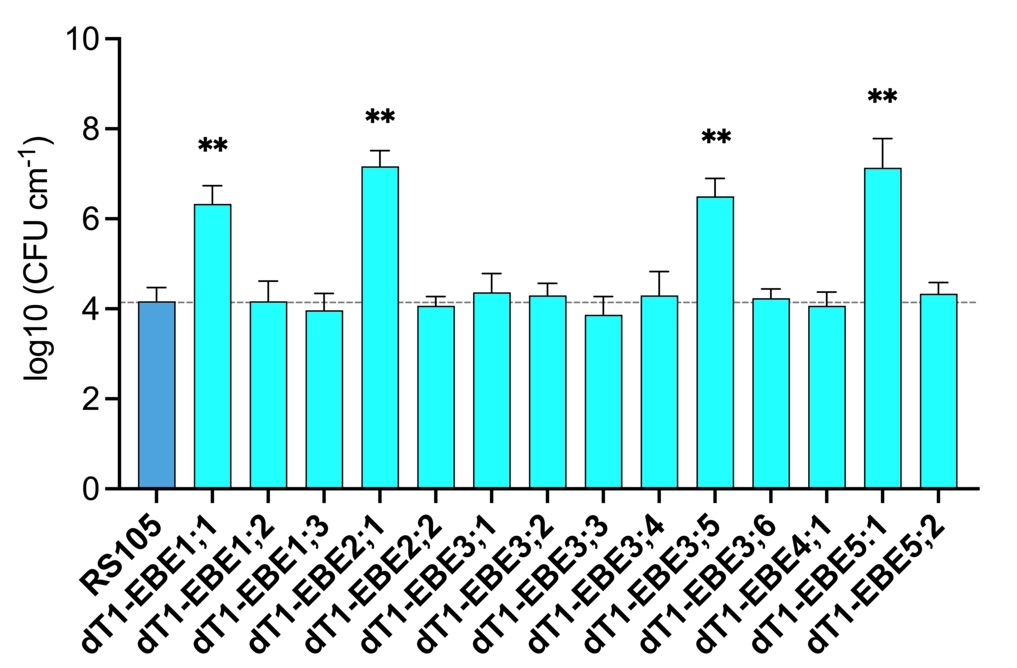
***inoculation 24 hpi.


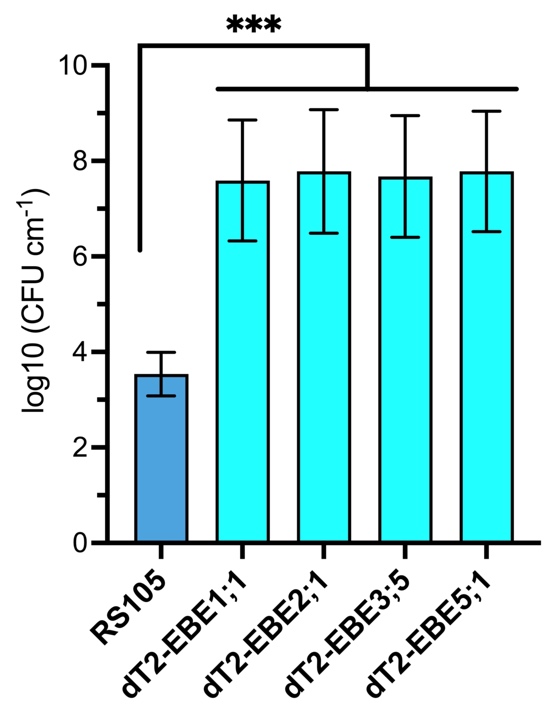
**Figure S4a.** The bacterial population count in ∆CDS-S1 mutant plant after inoculation of the first set of dTALE-RS105 strains. Samples were taken 7 days post-inoculation. Inoculation was performed with *Xoc* strain RS105.

**Figure S4b.** The bacterial population counts in ∆the CDS-S1mutant plant after inoculation of the second set of dTALE-RS105 strains. Samples were taken in two-day intervals for the first 10 days and then on the 14^th^ day post-inoculation. Inoculation was performed with *Xoc* strain RS105.

**
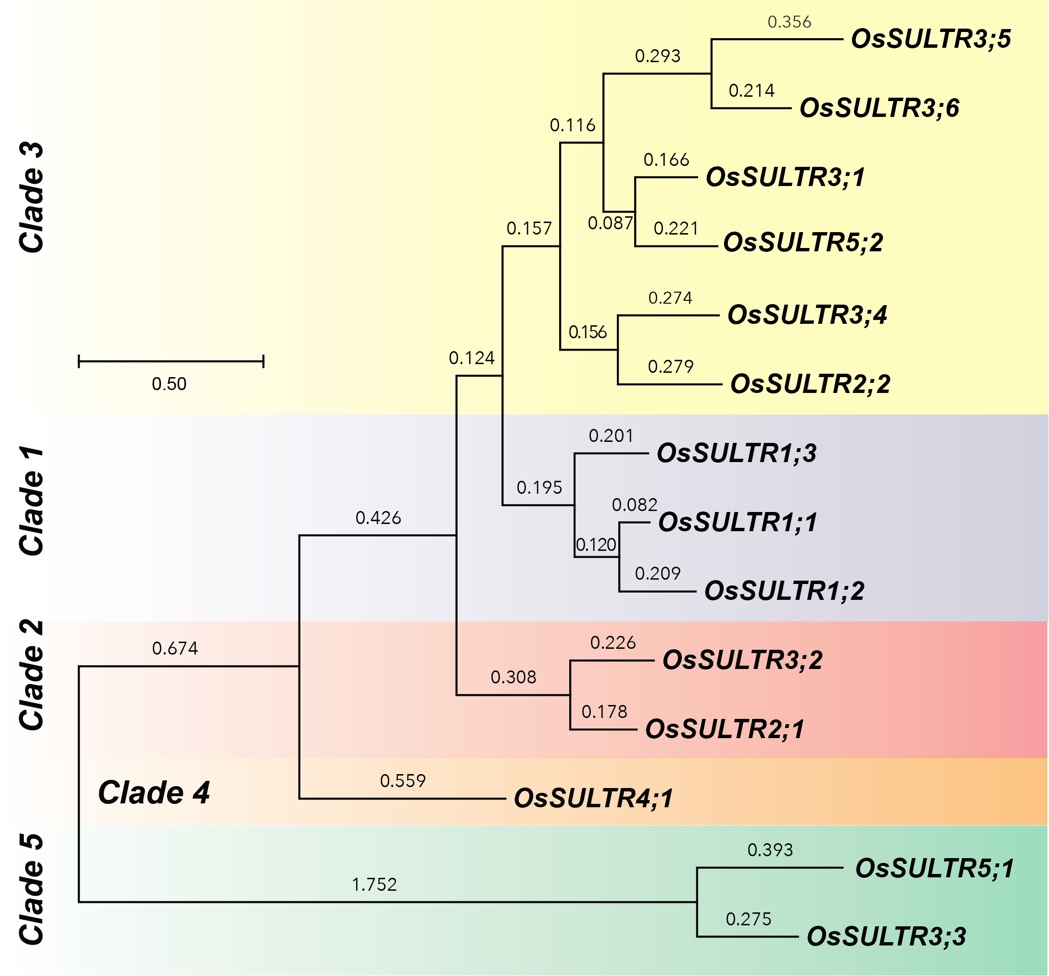
Figure S5.** Phylogenetic tree of SULTRs proteins family in Rice based on a neighbor-joining method using MEGA11. *OsSULTR5;1* and *OsSULTR5;2* were recently characterized as *OsMOT1;1* and *OsMOT1;2* respectively. Nomenclature was adapted from (Buchner et al., 2010).

**Table S1:** Cultivar IDs, subpopulation, and locations of cultivars showing ∆EBE_TalBF_ natural variation.

| **Cultivar Name** | **Cultivar ID** | **Subpopulation** | **Location** | **Accession** |
| --- | --- | --- | --- | --- |
| ANG_KANG_K.P. | IRIS_313-10898 | Indica III | Cambodia | ERS469742 |
| ATT_CHHMOUS | IRIS_313-12142 | Indica III | Cambodia | ERS469234 |
| DAMNOEUB_KRACHAK_SESS | IRIS_313-12151 | Indica III | Cambodia | ERS469240 |
| KAUN_TREI | IRIS_313-11999 | Indica III | Cambodia | ERS469138 |
| KREM_KRAHAM | IRIS_313-10904 | Indica III | Cambodia | ERS469702 |
| MUOY_KUM | IRIS_313-12038 | Indica III | Cambodia | ERS469169 |
| NEANG_CHEK | IRIS_313-12096 | Indica III | Cambodia | ERS469211 |
| NEANG_MAC | IRIS_313-10910 | Indica III | Cambodia | ERS469708 |
| NEANG_NARY | IRIS_313-10911 | Indica III | Cambodia | ERS469709 |
| RUS_RUSSEI | IRIS_313-12251 | Indica III | Cambodia | ERS469281 |
| Srau_Khgnaerng | IRIS_313-12143 | Indica III | Cambodia | ERS469235 |
| RPW9-4(SS1) | IRIS_313-9522 | Indica Intermediate | India | ERS468079 |
| BANDANG_PUTIH_680 | IRIS_313-10525 | Indica III | Indonesia | ERS469437 |
| DUD_KUNING | IRIS_313-9533 | Indica III | Indonesia | ERS468080 |
| IE_MATA_CICEM | IRIS_313-8978 | Indica III | Indonesia | ERS468272 |
| KEDOT | IRIS_313-8291 | Indica III | Indonesia | ERS468236 |
| MELABOH | IRIS_313-8493 | Indica III | Indonesia | ERS468246 |
| PADI_SIRANDAH_KUNING | IRIS_313-11904 | Indica III | Indonesia | ERS469047 |
| SI_OMPAN_LAMA | IRIS_313-11179 | Indica III | Indonesia | ERS470035 |
| CHAO_DO | IRIS_313-12188 | Indica III | Laos | ERS469252 |
| Khao_pa | IRIS_313-12130 | Indica III | Laos | ERS469224 |
| HAJI_HARUN_38 | IRIS_313-10694 | Indica III | Malaysia | ERS469510 |
| Innmayebaw | CX71 | Indica III | Malaysia | ERS470724 |
| PULUT_MERAH_2 | IRIS_313-11591 | Intermediate | Malaysia | ERS468780 |
| A_28-6 | IRIS_313-11406 | Indica III | Myanmar | ERS470198 |
| KYUN_TAW_SEIN | IRIS_313-11543 | Indica III | Myanmar | ERS468742 |
| THEEDAT_NGASEIN | IRIS_313-11146 | Indica III | Myanmar | ERS469946 |

**Table S1 (continued)**

| **Cultivar Name** | **Cultivar ID** | **Subpopulation** | **Location** | **Accession** |
| --- | --- | --- | --- | --- |
| YEBAWYIN | IRIS_313-8697 | Indica III | Myanmar | ERS467965 |
| ZEINGYI | IRIS_313-11150 | Indica III | Myanmar | ERS469951 |
| RAMINAD(GERMAN) | IRIS_313-11485 | Indica III | Philippines | ERS468696 |
| QUERO_ASSAN | IRIS_313-9898 | Indica III | Portugal | ERS468124 |
| NALUMOOLAI_KARUPAN | IRIS_313-9809 | Indica III | Sri Lanka | ERS467828 |
| KHAO_BUN_MI | IRIS_313-11680 | Indica III | Thailand | ERS468855 |
| KHAO_PRAHJIN | IRIS_313-8702 | Indica III | Thailand | ERS468255 |
| KHAO_SAHKORN | IRIS_313-11707 | Indica III | Thailand | ERS468944 |
| LEUANG_AWN | IRIS_313-8785 | Indica III | Thailand | ERS468265 |
| LEUANG_LAI_MAE_PRAJAN | IRIS_313-9209 | Indica III | Thailand | ERS468039 |
| LEUANG_TAWNG_SOOK | IRIS_313-9286 | Indica III | Thailand | ERS468052 |
| LEUM_DERN | IRIS_313-11838 | Indica III | Thailand | ERS468990 |
| NAM_SA-GUI_19 | IRIS_313-8485 | Indica III | Thailand | ERS467947 |
| NIAW_KHIAW_NGOO | IRIS_313-11683 | Indica III | Thailand | ERS468858 |
| OB_CHUEY | IRIS_313-11684 | Indica III | Thailand | ERS468859 |
| PAHK_NOK_GAEW | IRIS_313-9415 | Indica III | Thailand | ERS468067 |
| PAWNG_AEW_1 | IRIS_313-11508 | Indica III | Thailand | ERS468716 |
| RD19 | IRIS_313-10147 | Indica III | Thailand | ERS468159 |
| TONG_MAH | IRIS_313-9288 | Indica III | Thailand | ERS468282 |
| OM_2517 | W313 | Indica II | Viet Nam | SRR1240116 |

**
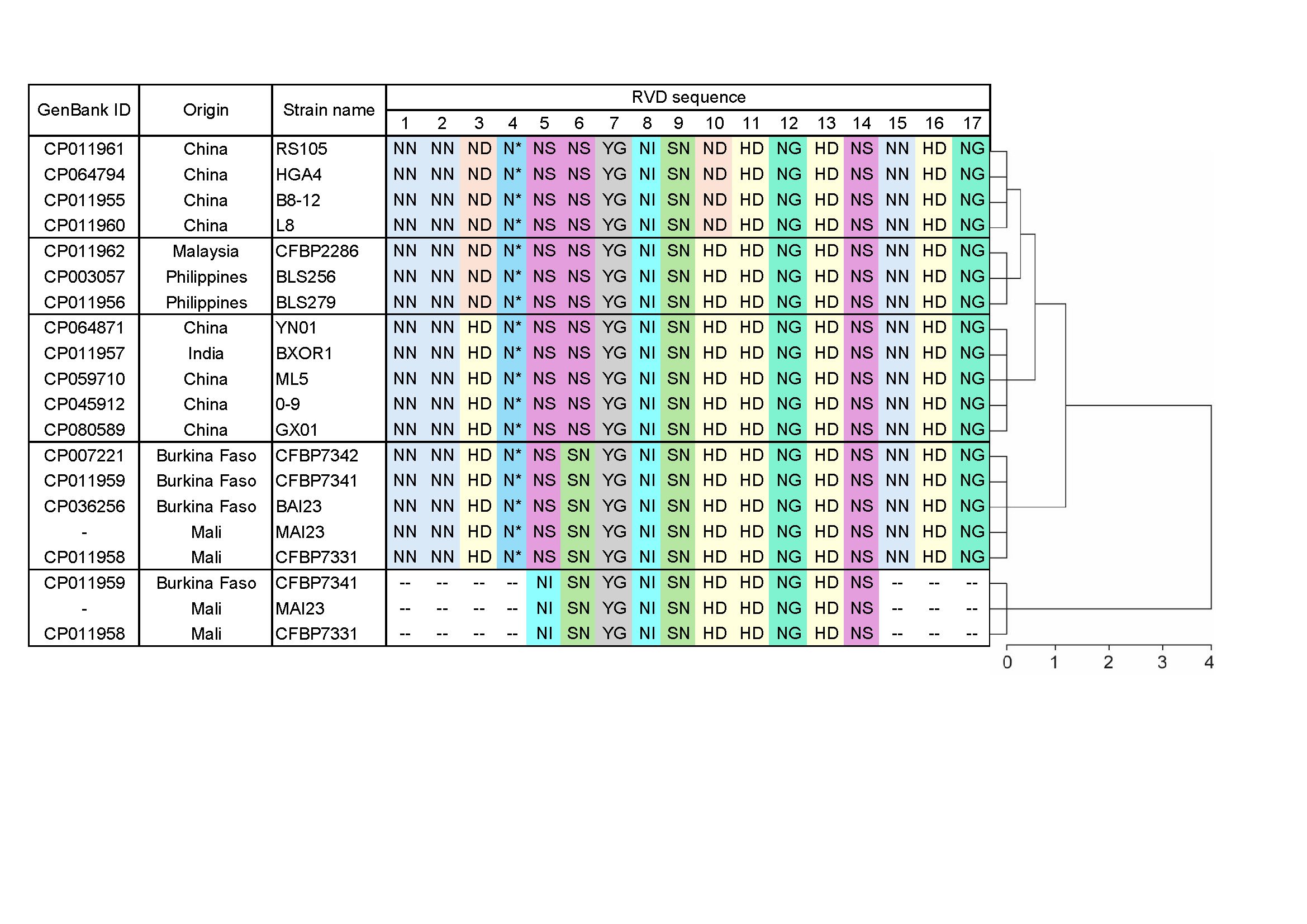
Table S2.** RVD sequence, alignment, and phylogenetic tree of TalBF family in whole genome sequenced strains of *Xoc.*

**
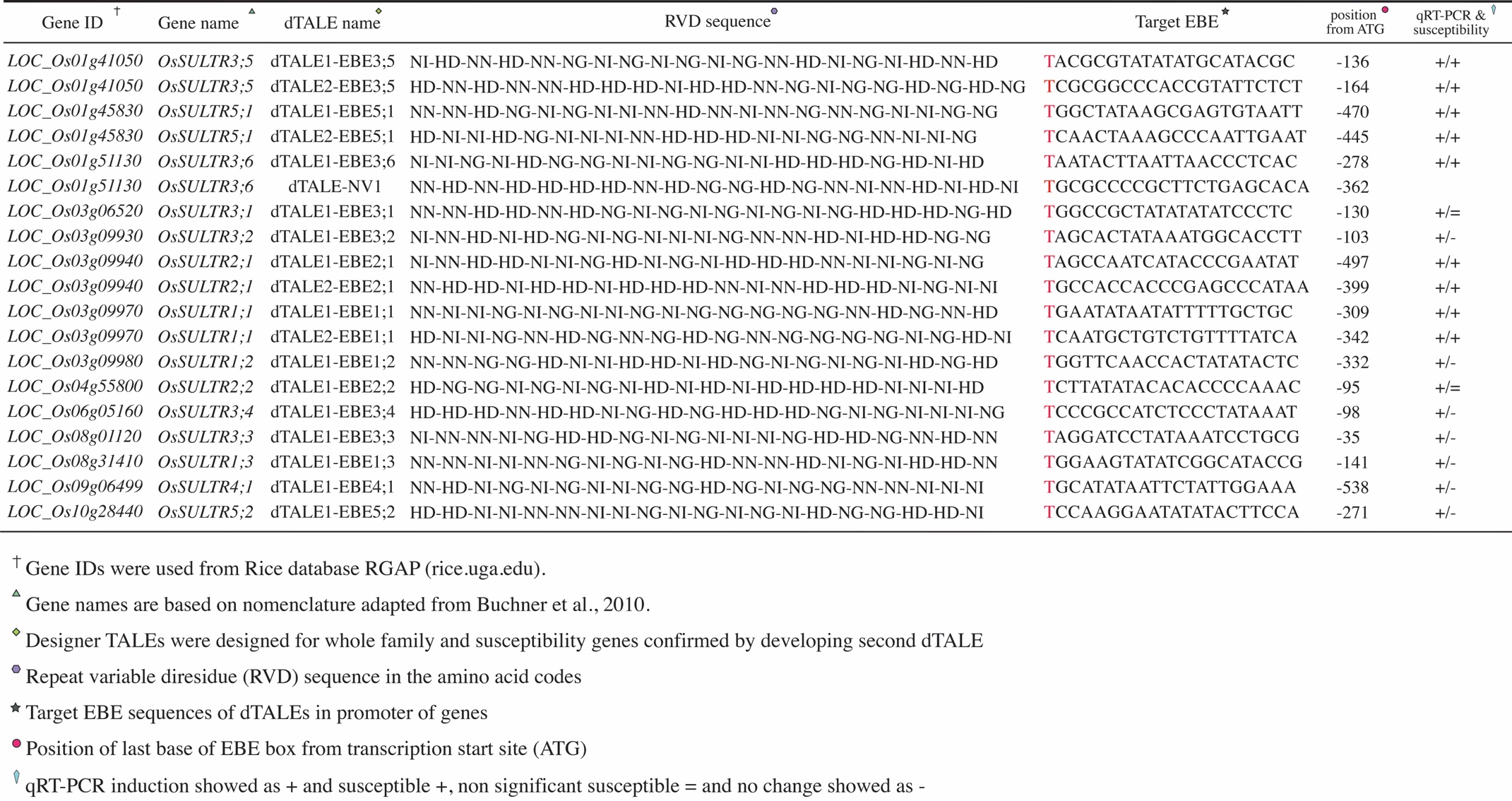
Table S3.** Sulfate transporter gene IDs, designer transcription activator-like effector (dTALE) repeat variable di-residue (RVDs), target sequences, and role in susceptibility.

**Table S4.** Primers used in the present study.

**Table S4a.** Primers used for qRT-PCR of dTALE-induced SULTRs genes

| **Gene ID** | **Gene Name** | **Primer Name** | **Primer Sequence 5' To 3'** |
| --- | --- | --- | --- |
| *LOC_Os01g41050* | *OsSULTR3;5* | qS35-F | GCATCAACCCGAGCTCGATA |
|  |  | qS35-R | CGTCGATGCGCTCTTTCTTC |
| *LOC_Os01g45830* | *OsSULTR5;1* | qS51-F | TCGTTCGTGATCCGTCCATC |
|  |  | qS51-R | GAAATTCATGAGCCCCACGC |
| *LOC_Os03g06520* | *OsSULTR3;1* | qS31-F | ATGAAGGAGACATTCCTGCC |
|  |  | qS31-R | CGAGCTTGGCGTAGCTGATG |
| *LOC_Os03g09930* | *OsSULTR3;2* | qS32-F | GTGACCTCATGGCTGGCCTAA |
|  |  | qS32-R | ACACAATGGCAACTGGTCCAATG |
| *LOC_Os03g09940* | *OsSULTR2;1* | qS21-F | TCAGGAGTGACGTCATGGCA |
|  |  | qS21-R | CCATGACGGCGTAGATCAGC |
| *LOC_Os03g09970* | *OsSULTR1;1* | qS11-F | CTCACCATTGCCAGCCTCTG |
|  |  | qS11-R | CCAGCGAAGAACGTTGCAGT |
| *LOC_Os03g09980* | *OsSULTR1;2* | qS12-F | GCCGAGGTCCAGGAAGCTAT |
|  |  | qS12-R | CTTGAGGTGCCCATCAGAGC |
| *LOC_Os04g55800* | *OsSULTR2;2* | qS22-F | CAAATGTGGCCTGAATCGCC |
|  |  | qS22-R | TGTGGTTCACAGCAGAACGA |
| *LOC_Os06g05160* | *OsSULTR3;4* | qS34-F | ATGCACTCCGTCTTCAAGCA |
|  |  | qS34-R | GGGTTCAATCCCTTGGGGAG |
| *LOC_Os08g01120* | *OsSULTR3;3* | qS33-F | GGCGTGGTAGTAGATCCGGA |
|  |  | qS33-R | GACAGCACGATGGGGATGTA |
| *LOC_Os08g31410* | *OsSULTR1;3* | qS13-F | TCACTCAAGCAGCCCTAGGA |
|  |  | qS13-R | CACCCGTGATGAGCTGAAGT |
| *LOC_Os09g06499* | *OsSULTR4;1* | qS41-F | TGGATGCGGACGTACAGGTT |
|  |  | qS41-R | GCCAACTGTCTCGATGAGCCA |
| *LOC_Os10g28440* | *OsSULTR5;2* | qS52-F | CATGGCATCCCGGTGATTGG |
|  |  | qS52-R | ATTGCGAAGCTCCTCCCGAT |
| *LOC_Os01g52130* | *OsSULTR3;6* | qS36-F | TACCGGCACGAGTGGAAATG |
|  |  | qS36-R | CCTCCGATGACGACAACCAT |

| **Primer Name** | **Sequence** | **Description** |
| --- | --- | --- |
| SULT-CpF2 | GATGATTAGCCGTACGTGAAACG | Used for genotyping of mutant plants |
| SULTR-CPR1 | ACAGAGGCCGATGATTGGAG |  |
| ∆SULTR-PseqF | GCCCAAACTACAACTTTCTCG | Used for promoter mutant plant genotyping |
| ∆SULTR-PseqR | GCATGCAGACAGAGAGAGAGTG |  |
| SULTR-OsU6aT1F | gccgACGTCGACGGCCGCAACCAC | CRISPR/Cas9 primers for sgRNA target ligation to sgRNA cassette |
| SULTR-OsU6aT2F | gccgACCACAGCTTCTCCGCGAAC |  |
| SULTRP-OsU6aT1F | gccgAGGAGATCGAACGGTGCCTT | CRISPR/Cas9 primers for sgRNA target ligation to sgRNA cassette |
| SULTRP-OsU6aT2F | gccgCGCATGGCGCGGTTTATAT |  |
| SUL-SNPF2 | TACTGTAGCCACTGCTCGCT | Used for genotyping of natural variation haplotype cultivars |
| SUL-SNPR2 | AGCAAAAGAACGACCCAACTT |  |
| SUL-SNPF3 | CAGACGTGTAGCACACTAGCA | Used for genotyping of natural variation haplotype cultivars |
| SUL-SNPR3 | AAATGCACAGCCAAGGGGA |  |
| SUL-SNPF4 | TCTTGGTGGTATGACTGTCTGAAT | Used for genotyping of natural variation haplotype cultivars |
| SUL-SNPR4 | TCTGGATATCTGCAGCGCGT |  |
| SUL-SNPF2-2 | TCGGTGCTCGCGCGTAATACTTAAT | Used for genotyping of natural variation haplotype cultivars |
| SUL-SNPR2-2 | TGGAGAAGCAAAAGAACGACCCAACT |  |
| SUL-SNPF2-3 | CTCACACTACTGTAGCCACTGCTCG | Used for genotyping of natural variation haplotype cultivars |
| SUL-SNPR2-3 | ACGTCCAGAAAACACGGGAGAAGTA |  |
| OsActin-F | GTTCCTGCTGTTTGTTCTGTTG | Used amplification of Actin gene as internal in qRT-PCR |
| OsActin-R | ATCTCACGCATTACCCTACCTT |  |

**Table S4b.** Primers used for genotyping of mutant and natural variation accessions.

**Table S5a.** Genetic diversity index, CDS haplotype information, and diversity of *OsSULTR3;6* in 3KRG. The data shows that this gene is relatively conservative and has low diversity.

| **Gene ID** | **Shannon’s equitability (*E_H_*)** | | | | | | | | | | | | | | | | | | | | | | | | | |  |  |
| --- | --- | --- | --- | --- | --- | --- | --- | --- | --- | --- | --- | --- | --- | --- | --- | --- | --- | --- | --- | --- | --- | --- | --- | --- | --- | --- | --- | --- |
|  | **3KRG** | ***Aus*** | ***Bas*** | **Adm** | ***XI*** | | ***XI* subpopulation** | | | | | | | | | | ***GJ*** | | ***GJ* subpopulation** | | | | | | | | | |
|  |  |  |  |  |  |  | ***XI-1A*** | | ***XI-1B*** | | ***XI-2*** | | ***XI-3*** | | ***XI-adm*** | |  | | | ***GJ-tmp*** | | ***GJ-sbtrp*** | | ***GJ-trp*** | | ***GJ-adm*** | |  |
| *LOC_Os01g52130* | 0.198 | 0.142 | 0.529 | 0.461 | 0.085 | | 0.062 | | 0.068 | | 0.084 | | 0.093 | | 0.116 | | 0.233 | | | 0.093 | | 0.114 | | 0.320 | | 0.221 | |  |
| *XI*, *Xian/indica*; *GJ*, *Geng/japonica*; *Bas*, *Basmati*; *Adm*, *Admixtures.* | | | | | |  | |  | |  | |  | |  | |  | |  | | |  | |  | |  | |  |  |

**Table S5b.** Tajima’s D index for *LOC_Os01g52130* gene in 3KRG subpopulations. This gene found to have been selectively had a distinct genotype in indica rice. Additionally, this differentiation is primarily evident in wild rice. This implies that the high-frequency alleles found in indica rice may directly originate from *O. nivara*. However, the situation is somewhat more intricate in the case of japonica rice. At least, the predominant high-frequency alleles in tropical japonica rice can be traced back to *O. rufipogon*.

| **Gene** | **3KRG** | **XI** | **GJ** | **XI-1A** | **XI-1B** | **XI-2** | **XI-3** | **XI-adm** | **GJ-adm** | **GJ-sbtrp** | **GJ-trp** | **GJ-tmp** | **Aus** | **Bas** | **adm** |
| --- | --- | --- | --- | --- | --- | --- | --- | --- | --- | --- | --- | --- | --- | --- | --- |
| **Tajima’s D** | 1.39363 | -1.40139 | 2.12523 | -0.55043 | -2.4187 | -1.4331 | -1.4674 | -1.4170 | -0.7950 | -1.7662 | 1.8052 | -2.29099 | 0.13249 | 2.0657 | 1.4691 |

**Table S6.** Table of plasmids and bacterial strains used in the study

| Bacteria | Description | origin |
| --- | --- | --- |
| E. coli (Top10') | *Escherichia coli* strain for culturing plasmid | This lab |
| *A. tumefences* (EHA105) | Agrobacterium strain used for transformation of CRISPR/Cas9 construct to rice calli | This lab |
| RS105 | Chinese strain of *Xoc* | This lab |
| BLS256 | The Philippine strain of *Xoc* | This lab |
| **Plasmids** |  |  |
| pYL-CRISPR/Cas9 | CRISPR/Cas9 vector for targeted mutations in plants | This study |
| pD-TALE-Repeats | The central repeat unit of pdTALE plasmid | This study |
| pKSS-Avr∆Xa23 | Activation domain vector of dTALE | This study |
| pHM1 | Cosmid used for transformation of pdTALE | This study |
| dT-NV1 | dTALE construct inducing *OsSULTR3;6* expression in ∆EBETalBf plants | This study |
| pHM1-pHWZ-dT1-E1;1 | First dTALE construct inducing *OsSULTR1;1* expression | This study |
| pHM1-pHWZ-dT2-E1;1 | Second dTALE construct inducing *OsSULTR1;1* expression | This study |
| pHM1-pHWZ-dT1-E1;2 | dTALE construct inducing *OsSULTR1;2* expression | This study |
| pHM1-pHWZ-dT1-E1;3 | dTALE construct inducing *OsSULTR1;3* expression | This study |
| pHM1-pHWZ-dT1-E2;1 | dTALE construct inducing *OsSULTR2;1* expression | This study |
| pHM1-pHWZ-dT2-E2;1 | Second dTALE construct inducing *OsSULTR2;1* expression | This study |
| pHM1-pHWZ-dT1-E2;2 | dTALE construct inducing *OsSULTR2;2* expression | This study |
| pHM1-pHWZ-dT1-E3;1 | dTALE construct inducing *OsSULTR3;1* expression | This study |
| pHM1-pHWZ-dT1-E3;2 | dTALE construct inducing *OsSULTR3;2* expression | This study |
| pHM1-pHWZ-dT1-E3;3 | dTALE construct inducing *OsSULTR3;3* expression | This study |
| pHM1-pHWZ-dT1-E3;4 | dTALE construct inducing *OsSULTR3;4* expression | This study |
| pHM1-pHWZ-dT1-E3;5 | dTALE construct inducing *OsSULTR3;5* expression | This study |
| pHM1-pHWZ-dT2-E3;5 | dTALE construct inducing *OsSULTR3;5* expression | This study |
| pHM1-pHWZ-dT1-E3;6 | dTALE construct inducing *OsSULTR3;6* expression | This study |
| pHM1-pHWZ-dT1-E4;1 | dTALE construct inducing *OsSULTR4;1* expression | This study |
| pHM1-pHWZ-dT1-E5;1 | dTALE construct inducing *OsSULTR5;1* expression | This study |
| pHM1-pHWZ-dT2-E5;1 | dTALE construct inducing *OsSULTR5;1* expression | This study |
| pHM1-pHWZ-dT1-E5;2 | dTALE construct inducing *OsSULTR5;2* expression | This study |

**References**

**Buchner, P., Parmar, S., Kriegel, A., Carpentier, M., and Hawkesford, M.J.** (2010). The sulfate transporter family in wheat: tissue-specific gene expression in relation to nutrition. Mol Plant **3**:374-389. 10.1093/mp/ssp119.
